# Mortalin, Apoptosis, and Neurodegeneration

**DOI:** 10.3390/biom2010143

**Published:** 2012-03-01

**Authors:** Carolina Londono, Cristina Osorio, Vivian Gama, Oscar Alzate

**Affiliations:** 1 Systems Proteomics Center Laboratory, School of Medicine, University of North Carolina, Chapel Hill, NC 27599; Escuela de Medicina, Universidad Pontificia Bolivariana, Medellín, Colombia; Email: londonop@email.unc.edu; 2 Systems Proteomics Center Laboratory and Program in Molecular Biology and Biotechnology, School of Medicine, University of North Carolina, Chapel Hill, NC 27599, USA; Email: osorioc@email.unc.edu; 3 Neuroscience Center, School of Medicine, University of North Carolina, Chapel Hill, NC 27599, USA; Email: vivian_gama@med.unc.edu; 4 Systems Proteomics Center Laboratory, Department of Cell and Developmental Biology, Program in Molecular Biology and Biotechnology and Department of Neurology, School of Medicine, University of North Carolina, Chapel Hill, NC 27599; Escuela de Medicina, Universidad Pontificia Bolivariana, Medellin, Colombia

**Keywords:** Alzheimer’s disease, apoptosis, GRP75, mortalin, mtHsp70, neurodegeneration, oxidative stress, Quantitative Intact Proteomics, p53

## Abstract

Mortalin is a highly conserved heat-shock chaperone usually found in multiple subcellular locations. It has several binding partners and has been implicated in various functions ranging from stress response, control of cell proliferation, and inhibition/prevention of apoptosis. The activity of this protein involves different structural and functional mechanisms, and minor alterations in its expression level may lead to serious biological consequences, including neurodegeneration. In this article we review the most current data associated with mortalin’s binding partners and how these protein-protein interactions may be implicated in apoptosis and neurodegeneration. A complete understanding of the molecular pathways in which mortalin is involved is important for the development of therapeutic strategies for cancer and neurodegenerative diseases.

## 1. Introduction

### 1.1. Mortalin: Structure and Known Functions

Mortalin is a 74 kDa mitochondrial-resident protein also known as p66mot-1 [1], mitochondrial stress-70 protein (mtHsp70) [2], peptide-binding protein 74 (PBP74) [3], and GRP75 [4]. Despite not being a heat-activated protein, based on sequence similarity, mortalin has been classified as another member of the heat shock protein 70 (Hsp70) family of chaperone proteins [5]. Mortalin, which is encoded by the nuclear gene HSPA9B (GeneID: 3313) [6,7,8], contains an N-terminal 46-amino-acid-long signal peptide that undergoes calcium-dependent autophosphorylation [9]. Genomic analysis revealed the presence of 2.8 kb human mortalin transcribed from an 18 kb region on chromosome 5q31.1 consisting of 17 exons with boundaries almost identical to its murine counterpart [10], and the first intron interrupted in the N-terminal leader sequence, a pattern similar to that of cytochrome-c (cyt-c), another mitochondrial protein [11].

**Figure 1:**
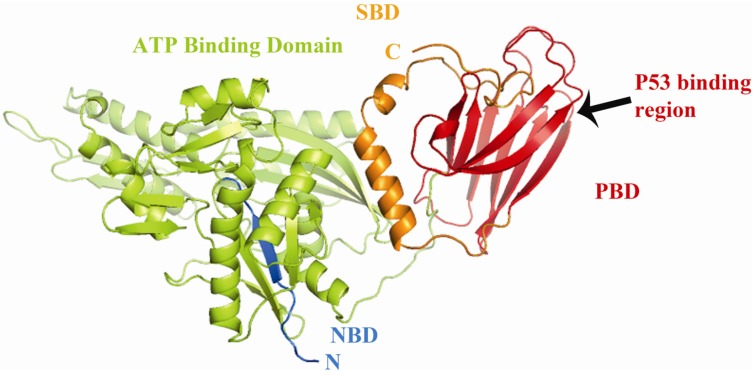
Molecular modeling of Mortalin. Representation of the 3D structure of mortalincreated by homology modeling with the program PyMOL (The PyMOL Molecular Graphics System [22]) and energy-minimized with Hyperchem 8.0 (Hypercube, Inc. Gainsville, FL. USA). The (N-terminal binding domain, **NBD**; amino acid residues 1–443) includes the N-terminal region (blue) and includes the ATP binding motif (amino acid residues 61–443; indicated in green); the substrate binding domain (**SBD**; amino acid residues 444–679) is shown in yellow and includes the peptide binding domain (**PBD**; amino acid residues 444–581, indicated in red) [2,5,12]. p53 binds mortalin somewhere in the peptide-binding domain of mortalin (black arrow) [23].

Mortalin is translated in the cytoplasm and is transported into mitochondria [12]. The crystal structure of mortalin has not yet been elucidated; therefore using amino acid sequence comparison and molecular modeling we developed a potential 3D structure (Figure 1). This 3D structural representation suggests that mortalin has two functional domains: an ATPase, N-terminal nucleotide-binding domain (NBD) and the C-terminal substrate-binding domain (SBD) [13]. The biochemical activities of each domain are essential for both general and specialized chaperone functions [14].

**Figure 2:**
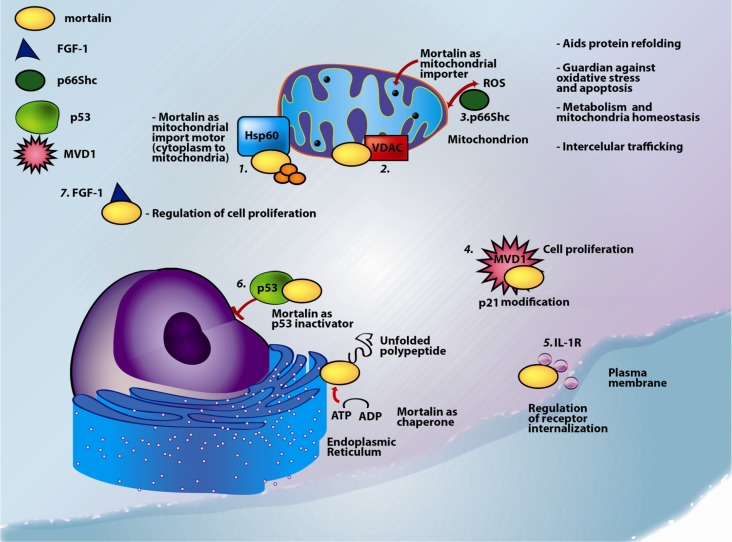
Multiple functions and multiple localizations of mortalin. Mortalin is involved in mitochondrial, nuclear, plasma membrane and endoplasmic reticulum processes. The distribution of mortalin is highly dependent on cellular conditions. Mortalin interacts with the following proteins: *1*. mitochondrial pre-proteins interact with mortalin and Hsp60 upon entering the mitochondrial matrix compartment; following these interactions, the mortalin/Hsp60 complex acts as a mitochondrial import motor. This coupling process allows proteins to refold, assemble, sort, and perform their corresponding functions; *2*. mortalin interacts with VDAC1 and modulates its channel properties; *3*. p66Shc localizes into the mitochondria and forms a complex with mortalin that modulates the mitochondrial pathway of apoptosis; *4*. binding of mortalin to MVD1 (that inhibits p21(ras)-induced growth arrest) may represent another pathway to immortalization and may be a part of mechanisms of cell proliferation; *5*. mortalin associates with the IL-1R (interleukin-1 receptor) protein leading to receptor internalization and downstream signaling cascades; *6*. mortalin binds p53 thereby inactivating p53 translocation to the nucleus and inhibiting its activity as an apoptosis inducer; and *7*. mortalin promotes intracellular trafficking of FGF-1.

Despite being predominantly localized in the mitochondria [1,15,16], mortalin has also been found in other sub-cellular compartments, including the endoplasmic reticulum [17], cytoplasmic vesicles [18], and the cytosol [2,17,19]. Mortalin activity and function are determined by its localization in the cell and by its binding partners (Table 1, and Figure 2). Several post-translational modifications (PTMs) have been found in mortalin, including phosphorylation, oxidation, and ubiquitination [19]. We found that mortalin is likely to be differentially phosphorylated in brain samples from Alzheimer’s disease patients [20], and that it is oxidized in the brains of h*APOE* targeted replacement mice [21]. Further confirmation of mortalin phosphorylation, identification of the specific phosphorylation sites, and elucidation of the biological effects of differential phosphorylation on mortalin function are still in progress.

Mortalin is a stress response protein induced by metabolic stress, glucose deprivation [24,25], the calcium ionophore A23187 [26], thyroid hormone treatment and hyperthyroidism [27], ionizing radiation [28] and some cytotoxins [19]. Increasing levels of mortalin expression are associated with cellular protection, as they permit cells to survive lethal conditions [29,30,31]. Mortalin has also anti-apoptotic [15] and pro-proliferative activities [32]. Mortalin accelerates the immortalization of normal human cells in cooperation with telomerase [33], and influences the function, dynamics, morphology, and homeostasis of mitochondria [15].

Depending on its localization and its binding partners, the following functions have been associated with mortalin: control of cell proliferation [34], intracellular trafficking [35,36], guidance of other proteins to their final localization [34], antigen processing [3,37], regulation of cell response to stress conditions [25,26,27,38], regulation of cell response to variation in glucose levels [25], receptor internalization and muscle activity [39], *in vivo* nephrotoxicity and cell fate determination [40], inactivation of the tumor suppressor protein p53 [34,41,42], and inhibition of apoptosis (programmed cell death) [32]. All of these functions and the corresponding binding partners are summarized in Table 1 and are represented in Figure 2.

**Table 1:** Proteins that bind or regulate mortalin and corresponding functions.

Protein	Subcellular location	Function	Reference
Amyloid precursor protein (APP)	Membrane. APP is an integral membrane protein expressed in many tissues and concentrated in the synapses of neurons.	Induces specific subsets of neuroprotective and anti-oxidative genes, mitochondrial regulatory genes and developmental genes.Activates mortalin expression	[43]
Apolipoprotein E (ApoE) - Evidence suggesting that ApoE binds mortalin is shown in Figure 4	Secreted	ApoE mediates the binding, internalization and catabolism of lipoprotein particles. It can serve as a ligand for the low density lipoprotein (ApoB/E) receptor and for the specific ApoE receptor (chylomicron remnant) of hepatic tissues.	See Figure 4
CDK11p60	CDK11p60 is the N-terminal portion of the cytosolic protein CDK11p110, that translocates into the mitochondria	Contributes to apoptosis directly at the mitochondria where it binds mortalin *in vivo* in cells undergoing Fas-induced apoptosis	[44]
Protein Dj-1	Predominantly cytoplasmic, nucleus, and mitochondria	Dj-1 protects cells against oxidative stress and cell death.Associated with Parkinson’s Disease.	[45,46,47]
Fibroblast growth factor 1 (FGF-1)	Nucleus, cytoplasm, cytosol, and cytoplasmic vesicles	FGF-1 is involved in the regulation of cell proliferation, differentiation, and migration.	[35,48]
94 kDa glucose-regulated protein (GRP94), tumor rejection antigen 1	Endoplasmic reticulum (ER)	GRP94 is a molecular chaperone that functions in the processing and transport of secreted proteins. Functions in ER-associated protein degradation.	[49]
Heat shock protein 60 kDa (Hsp60)	Mitochondrial matrix	Hsp60 is implicated in mitochondrial protein import and macromolecular assembly, including facilitating proper folding of mitochondrial imported proteins. May also prevent protein misfolding and promote the refolding and proper assembly of unfolded polypeptides generated under stress conditions in the mitochondrial matrix.	[9]
Hyaluronan-mediated motility receptor (RHAMM)	Centrosomes and microtubules, cytoplasmic	Involved in cell motility. When hyaluronan binds to HMMR, the phosphorylation of a number of proteins occurs. May also be involved in cellular transformation and metastasis formation, and in regulating extracellular-regulated kinase (ERK) activity.	[50]
Interleukin-1 (IL-1)-α receptor	Secreted	Major proinflammatory cytokine mediating local and systemic responses of the immune system.An important protein during neuroinflammation and neurodegeneration.	[36]
Diphosphomevalonate decarboxylase (MVD1); previously known as MPD	Cytosol	MVD1 is involved in cholesterol biosynthesis, providing prenyl groups required for protein prenylation.	[51]
p53	Cytosol, mitocondria	p53 is a tumor suppressor protein; it participates in apoptosis and genomic stability.	[23,52]
SHC-transforming protein 1 - p66 isoform, p66Shc	mitochondrion	The 66 kDa isoform of the SHC-transforming protein regulates lifespan in mammals, and is a critical component of the apoptotic response to oxidative stress.	[53,54]
NADH dehydrogenase	Mitochondrial inner membrane.	Core subunit of the mitochondrial membrane respiratory chain. NADH dehydrogenase - complex I, functions in the transfer of electrons from NADH to the respiratory chain.	[2]
E3 ubiquitin-protein ligase, Parkin	Mainly cytosolic, nucleus, ER, and mitochondria.	Parkin is involved in the regulation of mitochondrial morphology, antagonizing oxidative damage to mtDNA and activating mitochondrial self-repair mechanisms.	[15,55]
Tid1 (DnaJ (Hsp40) homolog, subfamily A, member 3)	Mitochondrial matrix	Nucleotide exchange factor.Heat shock protein co-chaperone.	[14,56]
TNF receptor-associated protein (TRAP-1)	Mitochondrial matrix	Chaperone, preserves mitochondrial membrane potential, maintains ATP levels and cell viability during stress.	[57]
Voltage-dependent anion-selective channel (VDAC)	Mitochondrial outer membrane, cell membrane	Participates in energy metabolism, mitochondrial homeostasis, and apoptosis. It also may participate in the formation of the permeability transition pore complex (PTPC) responsible for the release of mitochondrial products that triggers apoptosis.	[58]

### 1.2. Mortalin and Mitochondrial Function

Mortalin is involved in multiple basic mitochondrial processes, including energy metabolism, free-radical generation [31], and maintenance of mitochondrial protein integrity [19,59]. In addition, mortalin plays a role in mitochondrial biogenesis, translocation of cytosolic protein precursors, and their partitioning within the matrix and across the two mitochondrial membranes [23,60,61] (Figure 2). Mortalin is the only ATPase component of the pre-protein mitochondrial import machinery [62,63] where it binds the translocase of the mitochondrial inner membrane (TIM) to form an ATP-dependent motor [15,64,65]. In the mitochondrial matrix, mortalin floats freely as it participates in protein folding in association with Hsp60 [5].

**Figure 3:**
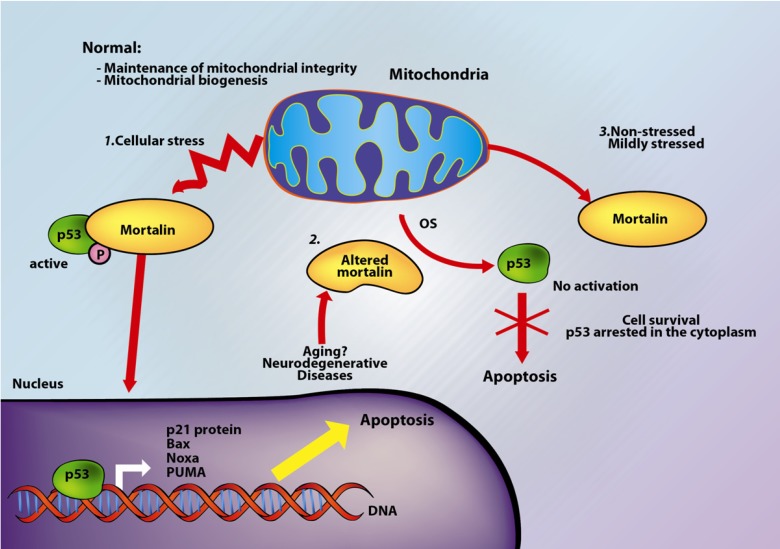
Role of mortalin in oxidative stress-induced apoptosis. Mortalin has different functions under cellular stress or under non-stressed conditions. *1*. Exposure of cells to stress induces the phosphorylation of p53 and its interaction with mortalin. Mortalin tries to protect the cells against oxidative damage; however, if the cells cannot recover, p53 induces the transcriptional activation of Bax, and BH3-only proteins including Noxa and PUMA, resulting in apoptosis; *2*. increased levels of cellular oxidative stress can alter mortalin’s function; *3*. In non-stressed (normal) or mildly-stressed conditions the phosphorylation levels of p53 are low, and mortalin does not interact with p53.

In eukaryotic cells the majority of mitochondrial precursor proteins (pre-proteins) are synthesized in the cytosol, recognized by receptor proteins on the mitochondrial surface, and translocated across the mitochondrial membranes [64,65] via specific transport machines. These machines include the Translocase of the Outer Membrane (TOM, [66]) and the Translocase of the Inner Membrane (TIM, [15,65,67]). These molecular machines are used to bring the translocated proteins to their final destination in the mitochondria, including the intramembrane space (IMS), the inner membrane, and the matrix [68,69,70]. Many mitochondrial precursor proteins are chaperoned into the mitochondrial matrix by mortalin, using an ATP-dependent mechanism and the assistance of co-chaperones [69,71]. Some specific details of the mortalin-associated translocation mechanism are shown in Figure 3 and include:

- Tim44, a matrix protein, associates simultaneously the Tim23 complex (the translocation channel in the inner membrane) and mortalin [72,73].
- Tim14 (Pam18 or DNAJC19), a J-domain protein, stimulates mortalin’s ATPase activity [74].
- Tim16 (Pam16 or Magmas), which controls the activity of Tim14 and Mge1 (hMge1), stimulates the release of adenosine diphospate (ADP) [14,73].

The matured protein is then transferred by mortalin to the Hsp60 protein. Hsp60 allows proteins to refold back, assemble, sort and finally perform their duties as components in the bioenergetics network [5]. This coupling is essential for maintaining the mitochondrial proteome integrity [19].

Mitochondria are vulnerable to oxidative damage, including oxidative stress (OS) in which free radicals modify proteins, lipids, and nucleic acids [75]. Under normal conditions, the mitochondrial electron transport results in production of reactive oxygen species (ROS) [31] that, in excess, can result in cellular membrane damage and cellular dysfunction [76]. ROS are a causal step in apoptosis and a key element in some neurodegenerative diseases [15]. The importance of mortalin in ROS-associated neurodegeneration stems from the fact that mortalin inhibits ROS accumulation in the mitochondria [24,45,77]. Glucose deprivation causes a rapid increase in ROS accumulation, which is reduced by mortalin over-expression, suggesting that mortalin has a cytoprotective effect and could decrease the ROS accumulation maintaining cell viability [24].

## 2. Mortalin and Apoptosis

In multicellular organisms, cells that are no longer needed are destroyed by a regulated process known as apoptosis [78,79,80]. Apoptosis is important for embryo development, tissue homeostasis, and regulation of the immune system as well as for the development of the nervous system [79,81]. Apoptosis may play a role in neurodegeneration and aging [80,82].

There are two apoptotic pathways in mammals; *i.e.*, the extrinsic and the intrinsic pathways [83]. Both of these pathways involve the activation of caspases—proteolytic proteins that cleave their target polypeptides at specific locations without degradation of the target protein [84,85], thus creating gain-of-function or loss-of-function events that generate the apoptotic phenotype [85].

The intrinsic, or Bcl-2-regulated, mitochondrial pathway is triggered in response to several cellular stressors. Bcl-2 homology 3 (BH3)-only members either directly activate the pro-apoptotic Bcl-2 family members Bax and Bak, or antagonize anti-apoptotic Bcl-2 family members [86,87]. Bax and Bak are thought to homo-oligomerize and form pores in the outer mitochondrial membrane thereby increasing mitochondrial outer-membrane permeabilization (MOMP), considered as the ‘point of no return’ in apoptosis signaling. The MOMP allows the efflux of multiple pro-apoptotic proteins from the mitochondrial intermembrane space including cyt-c [85]; as a result, second mitochondrial activators of caspases (Smac or DIABLO) can pass from the intermembrane space into the cytoplasm. In the cytosol, cyt-c interacts with an adaptor protein, the apoptotic protease-activating factor-1 (Apaf-1), a crucial step of the intrinsic pathway [88]. The interaction between cyt-c and Apaf-1 induces Apaf-1 conformational changes driven by dATP hydrolysis [89]. The resulting complex recruits and binds pro-caspase-9 to the caspase recruitment domain (CARD) of Apaf-1 [84]. Caspase-9 is activated in a dATP/ATP-dependent process and the resulting complex cleaves, and activates caspases 3 and 7 [84,89,90,91]. These proteins mediate the molecular signals leading to cell death through the selective proteolysis of key protein substrates.

p53 is a key tumor-suppressor protein that abolishes genetically-unstable cells by inducing cell cycle arrest or apoptosis through transcriptional regulation or by direct interaction with apoptotic proteins [92]. p53 can be inactivated by post-translational modifications, mutations [93], or as a result of sequestration by binding proteins [94,95,96]. p53 has been implicated in transcriptional activation of several proteins (such as Ras, p21, Bax, BH3-only proteins Noxa and PUMA (p53-up-regulated modulator of apoptosis), PIG3, Killer/DR5, CD95 (Fas), p53AIP1 and Perp) or repression of genes involved in apoptosis [97]. 

Some studies have reported functional interactions between p53 and mortalin in the cytoplasm [23,52,53,92,98,99], leading to the inhibition of the transcriptional activation of p53 and control of centrosome duplication functions [92,99]. Specifically, p53 presents two binding sites for mortalin, one in the C-terminal domain and the other in the p53-tetramerization (TET) domain [23]; any of these domains is sufficient for a mortalin-p53 binding interaction. This interaction occurs through the PBD of mortalin [23,99] (Figure 1).

A recent study indicates that the mortalin-p53 interaction causes inactivation of p53-mediated apoptosis depending on the cellular stress levels [92]. Specifically, stress-associated induction of mortalin expression protects the cells against the initial insult, improves cell recovery, and improves resistance to subsequent stress signals. Unstressed or mildly stressed cells do not display mortalin-p53 interaction [92] (Figure 3). Mortalin may prevent the entry of p53 to the nucleus by physical entrapment that leads to proteasomal degradation [52]. During the late G1 phase, mortalin localizes in the centrosome and represses the p53-dependent suppression of centrosome duplication [98]. p53 can induce Bax activation, leading to changes in the mitochondrial membrane permeabilization; however, in the absence of mortalin, there is nuclear accumulation of p53, concomitant with increased levels of Bax, suggesting that the low levels (or absence) of mortalin cause activation of the p53-Bax apoptosis pathway [92]. Mortalin may also be associated with cell immortalization via binding to diphosphomevalonate decarboxylase (MVD1; previously known as mevalonate pyrophosphate decarboxylase or MPD), an inhibitory protein of p21 (Ras). Furthermore, co-expression of the human telomerase reverse transcriptase (hTERT) with mortalin can avoid cell death [33].

Another important protein in the mortalin/p53/OS-associated molecular mechanisms is the 66 kDa isoform of the SHC-transforming protein 1 or p66Shc, a protein that mediates OS-induced apoptotic mechanisms [53]. p66Shc is a downstream target of p53 that predominantly exists in the cytoplasm and is translocated into the mitochondria in a process mediated by mortalin and prolyl isomerase 1 (Pin1) [100]. In mitochondria, following pro-apoptotic stimulation, p66Shc oxidizes cyt-c, producing H_2_O_2_, which promotes the opening of the mitochondrial permeability transition pore triggering apoptosis [53].

The literature review presented here suggests that mortalin participates in apoptosis by regulating proteins that are implicated in cellular stress response mechanisms. Under low levels of stress, mortalin acts as an anti-apoptotic protein by inactivating p53 [32,92], and interfering with the ability of cyt-c and Apaf-1 to trigger the recruitment of procaspase-9; on the other hand, under stress conditions, mortalin alters mitochondrial functions while cytoplasmic p53 can induce apoptotic signals (Figure 3). These opposing functions point out to a mortalin protein that may represent a sensitive marker of stressed cells and apoptotic function associated with p53 activity.

## 3. Mortalin and Neurodegeneration

Aging is a biological process characterized by a general and progressive deterioration in metabolic processes affecting tissues that exhibit a high rate of oxygen consumption, such as the brain [19]. Aging and neurodegeneration also affect the proteome. Oxidative protein damage results in protein aggregation, changes to secondary and tertiary structures, and loss of catalytic functions that may activate cell death-associated signal transduction pathways. Unfolded proteins have a strong tendency to form neurotoxic insoluble protein aggregates resulting in the impairment of the ubiquitin-proteasome degradation system, and suppression of the heat shock and OS response mechanisms [101]. The abnormal accumulation of unfolded and/or aggregated polypeptides usually leads to the loss of specific neuronal populations resulting in the onset and progression of several neurodegenerative diseases [34,77]. In general, the coupling of stress with impairment of the chaperone system can cause premature aging [19].

**Figure 4:**
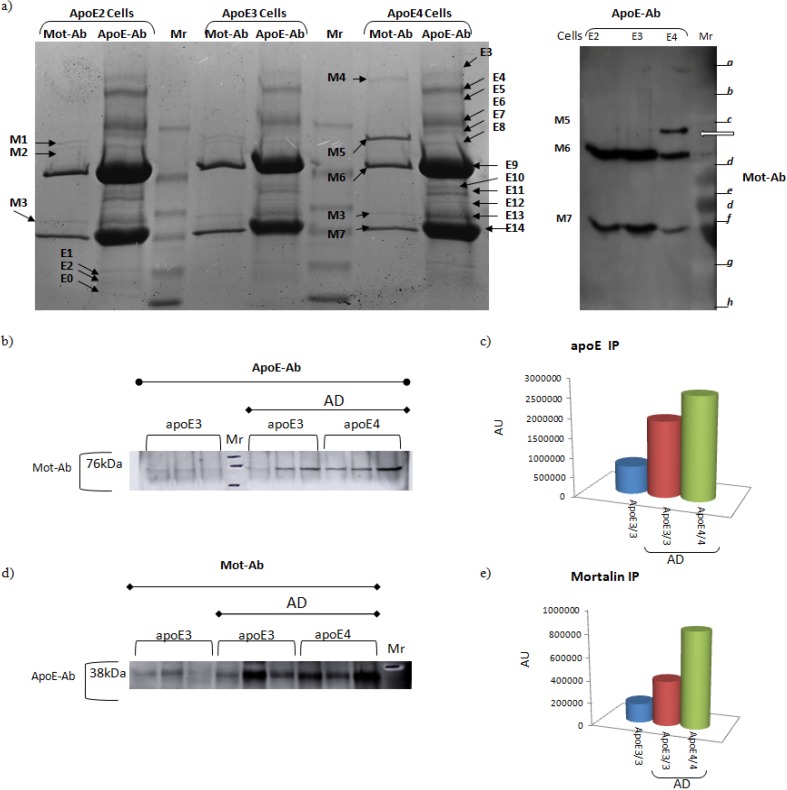
Potential interaction between mortalin and ApoE in Alzheimer’s disease. (**a**) Astrocytes from hApoE ε2/2-TR, hApoE ε3/3-TR, and hApoE ε4/4-TR mice were solubilized and immunoprecipitated with mortalin (Mot-Ab) or ApoE (ApoE-Ab) antibodies (Left panel). The ApoE-Ab immunoprecipitants were challenged with the Mot-Ab and only the hApoE ε4/4-TR astrocytes displayed interaction between ApoE and mortalin ((a) Right panel, indicated with a white arrow on the right panel). (**b**) Human brain tissues from hippocampus, were solubilized as described [20], followed by immunoprecipitation with an ApoE antibody. The immunoprecipitated proteins were separated in a 10% SDS-PAGE gel, transferred to a PVDF membrane, and immunoblotted with a Mot-Ab. Quantitation of the mortalin-apoE bands indicates that the binding is genotype- and disease-dependent (**c**). The complementary experiment, in which mortalin is immunoprecipitated with the Mot-Ab, and the IP is immunoblotted against ApoE-Ab (**d,e**) shows almost identical results. Proteins were identified by MALDI-TOF/TOF mass spectrometry (white arrow). “M” indicates proteins that were immunoprecipitated with the mortalin antibody and identified by mass spectrometry; correspondingly, “E” represents proteins that were immunoprecipitated with the apoE antibody.

The level of oxidized proteins in a cell reflects the balance between the rates of protein oxidation (generation of ROS) and protein degradation (degradation of oxidatively-damaged proteins) [76,102]. Some studies have shown that there is an association between aging and oxidative damage of stress chaperones [21], like mortalin, in neurodegenerative diseases, including Alzheimer’s disease [11,21,102,103] and Parkinson’s disease [15,104]. Our studies have demonstrated that mortalin is oxidized in the brain tissues of an animal model of Alzheimer’s disease [21]. Another potential role of mortalin in neurodegeneration stems from the participation of mortalin in calcium channel regulation [58], a critical process for neuronal health.

Apolipoprotein E (ApoE) is important in the regulation of cholesterol and metabolism of triglycerides. There are three common ApoE isoforms: ε2, ε3 and ε4. The *APOE*4 allele is associated with an increased risk of Alzheimer’s disease [105,106,107,108]. Studies of ApoE4 transgenic and ApoE-deficient mice have confirmed an association between reduced ApoE activity, oxidative damage, and age-dependent neuronal alterations. Using proteomics, Osorio *et al.* performed a study of human ApoE4-Targeted Replacement mice (hApoE4-TR) compared to hApoE3-TR as control [20]. It was found that different mortalin isoforms are present in hApoE4-TR and hApoE3-TR mice brains, as well as between Alzheimer’s disease patients and age- and gender-matched controls [20]. In addition, using immunoprecipitation with ApoE- and with mortalin-antibodies, we have found that mortalin binds ApoE in hApoE-TR mice, as well as in human brains of Alzheimer’s disease patients (Figure 4). This binding, whose functional effect is under investigation, is different between diseased and non-diseased brains, and between *APOE* ε3/3 and *APOE* ε4/4 genotypes (Figure 4).

OS, and mitochondrial and proteosomal dysfunction have been implicated in the pathogenesis of Parkinson’s disease [15,109,110,111]. Parkinson’s disease is a progressive disorder characterized by dopaminergic neurodegeneration in the *Substantia Nigra pars compacta* (SNpc) and by the appearance of proteinaceous cytoplasmic inclusions (Lewy bodies) in the remaining nigral neurons [15,112]. A reduced expression level of mortalin has been observed in the affected brain regions of Parkinson’s disease patients [15,113] and in a cellular model of Parkinson’s disease [47]. Specifically, in dopaminergic neurons, manipulations of the level of mortalin resulted in changes to the sensitivity to Parkinson’s disease phenotypes via different pathways related to OS, mitochondrial and proteasomal function [47], correlating with reduced mitochondrial membrane potential and increased production of ROS [45].

Like in other neurodegenerative diseases, ROS is a key element in the pathophysiology of Parkinson’s disease [114]. Parkin, an E3 ubiquitin–protein ligase that mediates polyubiquitination of VDAC [115], is associated with mitochondrial dynamics [116], is involved in the regulation of mitochondria morphology, and is related with autosomal-recessive Parkinson’s disease. Parkin may also play a role in sporadic cases of Parkinson’s disease. There is evidence indicating that mortalin and Parkin provide a protective effect against oxidative damage, and that mortalin is involved in Parkinson’s disease-related abnormal mitochondrial morphology. Under OS, mortalin knockdown stimulates disintegration of mitochondrial connectivity and low-grade branching of mitochondria [15].

Dj-1 is an oncogene that protects cells against OS and cell death, and mutations in Dj-1 are associated with familial forms of Parkinson’s disease [117]. Dj-1 is associated with chaperones including Hsp70, CHIP and mortalin and undergoes OS-mediated translocation into mitochondria [118]. Mortalin has been identified as one of the five major proteins (mortalin, nucleolin, Grp94, calnexin and clathrin) that bind α-synuclein and Dj-1, two critical proteins in Parkinson’s disease pathogenesis [34,47].

Mortalin-null cells exposed to OS show disintegration of mitochondrial connectivity, suggesting that mortalin is implicated in the control of the mitochondrial dynamics and morphology [15]. It has also been reported that Tid-1, a chaperone protein involved in the regulation of cell survival, interacts with mortalin on an isoform-specific basis, and can mediate the reactivation of protein aggregates. It was suggested that mortalin can serve as a scavenger of toxic protein conformers in human mitochondria, making it an attractive target for therapies against protein conformational diseases [14]. 

## 4. Mortalin, Apoptosis and Neurodegeneration

**Figure 5:**
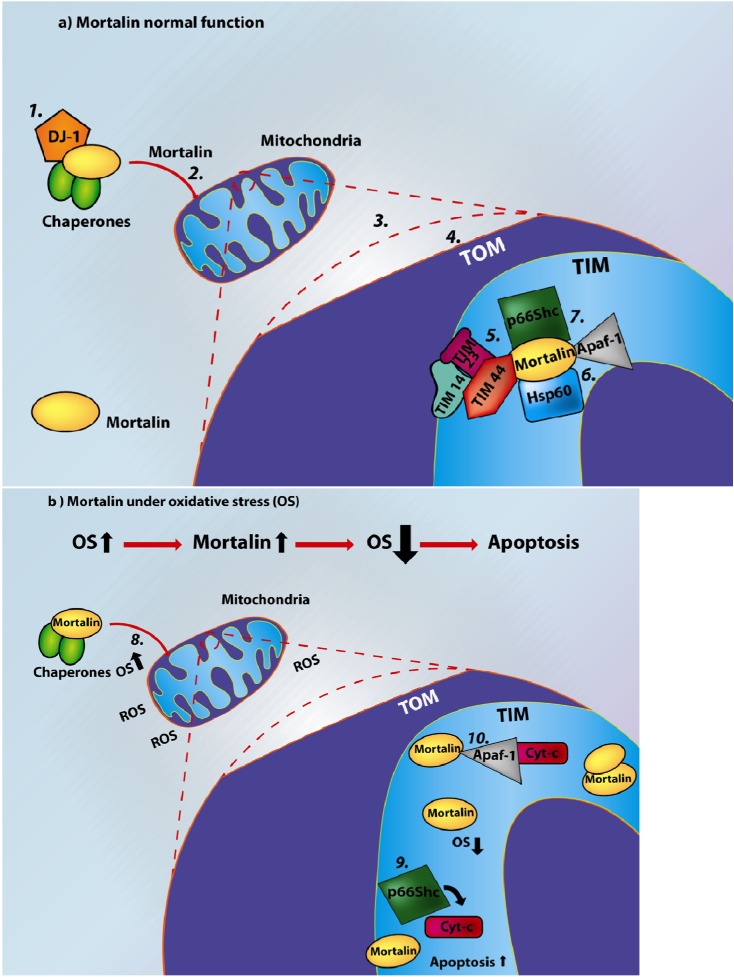
A schematic model showing the mechanism of action of mortalin. (**a**) The normal function of mortalin. Under normal conditions, mortalin associates with certain chaperones, including Dj-1 (*1* in the figure); following this interaction, the complex travels to the mitochondria, where mortalin is detached and enters the mitochondria (*2*). A magnified image (*3*) shows that mortalin crosses the outer membrane (TOM) (*4*) and the inner membrane (TIM) where it performs multiple functions, including chaperoning of the precursor proteins into the mitochondrial matrix (*5*). During, this process, mortalin binds simultaneously to TIM 44 (a peripheral membrane protein) and to the Tim23 complex (*5*). Next, the mature protein is transferred by mortalin to Hsp60 (*6*). Under normal conditions, mitochondrial mortalin forms stable complexes with p66Shc and Apaf-1 (*7*), which are released under cellular stress. (**b**) Under OS, mortalin levels are increased in the mitochondria, inhibiting ROS accumulation, and acting as a cytoprotective protein while maintaining cell viability (*8*). The magnified portion of the image shows that under stress conditions however, thelevels of mortalin needed to control other activities are reduced and the cells can suffer an imbalance; for example, the interaction between mortalin and p66Shc can be disrupted, and p66Shc oxidizes cyt-c and promotes the opening of the mitochondrial permeability transition pore triggering apoptosis (*9*); alternatively, Apaf-1 can induce apoptotic pathways (*10*). As a consequence, apoptosis increases in the absence of free mortalin, as a result of a rich OS environment.

Apoptosis allows the elimination of non-viable cells without affecting the neighboring cells. Unlike the rapid turnover of cells in proliferative tissues, neurons show only slight regeneration and normally stay alive for the entire life of the organism [119,120]. OS and metabolic stress are able to activate the chaperone system and can initiate neuronal apoptosis (Figure 5), and under OS there is inhibition of the electron transport chain and production of ROS in neurons [121]. Qu *et al.* demonstrated that overexpression of mortalin in neuroblastoma cells can reduce OS [30]. Mortalin increases the stress response capacity of the cells resulting in increased cell viability and extended longevity of an organism.

Mortalin responds to ROS accumulation under stress conditions while regulating other housekeeping functions, including control of cell proliferation, intracellular trafficking, or anti-apoptotic activity; this down-regulation of housekeeping functions may result in uncontrolled cell proliferation. For example, OS induces mortalin translocation into the mitochondria [104], leaving other proteins, including Apaf-1 and p53, unchecked, thus potentiating the disproportionate activation of apoptotic biochemical cascades.

From our observations that the mitochondrial proteome is affected by mortalin expression levels, we would expect that, under normal conditions, mortalin behaves as an anti-apoptotic protein that inactivates p53, resulting in cyt-c or Apaf-1 not being released. On the other hand, in the presence of oxidative stress, mortalin is responsible for mitochondrial homeostasis, allowing cytoplasmic p53 to induce apoptosis. These observations point to mortalin being a sensitive marker of stressed cells and the apoptotic function associated with p53 activity. Mortalin behaves as a regulatory protein that can alter cell function by associating with vital cellular proteins, including p53, Dj-1, FGF-1, and Hsp60. Regulating the functions of these proteins most likely affects signals involved in neurodegenerative diseases and apoptosis as discussed above. Bearing in mind the multiple functions of mortalin in cell control, is not surprising that over-expression of mortalin is able to promote cancer and may trigger features associated with neurodegenerative diseases, including Parkinson’s and Alzheimer’s diseases.

## 5. Concluding Remarks

Cellular homeostasis is maintained by a strict regulation of the balance between ROS production, cell growth, and apoptosis. Many pathological states, including cancer and neurological diseases, are often associated with OS and disregulation of apoptotic pathways. Changes in mortalin expression are associated with cellular protection as they permit cells to survive lethal conditions modulating the cell’s lifespan [29,30,31]. Mortalin also has anti-apoptotic and pro-proliferative activities that influence the functions, dynamics, morphology and homeostasis of mitochondria.

Proteomic, molecular and biochemical data suggest that cell death, degeneration, and immortalization are not controlled by a single mechanism; they are regulated by a complex networks of proteins interconnected via multiple molecular pathways. The mitochondria and mitochondrial proteins play a fundamental role and are an indispensable part of this regulatory network. Several process that are related to the mitochondria, such as apoptosis, have been extensively studied for many years, but some of the processes, such as protein import, complex assembly, and the molecular mechanisms by which mortalin influences apoptosis have not yet been sufficiently elucidated. This review summarizes the present knowledge on mortalin and its relationship to apoptosis and neurodegenerative diseases, and mortalin’s role in OS and mitochondrial function. Although several cellular proteins are known to interact with mortalin, mortalin appears to be a regulatory protein that maintains the integrity of the cell via multiple molecular processes that are still under investigation. Further research on the function and dynamics of mortalin could provide valuable information about the complex balance between longevity, neurodegeneration, and apoptosis.

## References

[1] Wadhwa R., Kaul S.C., Ikawa Y., Sugimoto Y. (1993). Identification of a novel member of mouse hsp70 family. Its association with cellular mortal phenotype. J. Biol. Chem..

[2] Bhattacharyya T., Karnezis A.N., Murphy S.P., Hoang T., Freeman B.C., Phillips B., Morimoto R.I. (1995). Cloning and subcellular localization of human mitochondrial hsp70. J. Biol. Chem..

[3] Domanico S.Z., DeNagel D.C., Dahlseid J.N., Green J.M., Pierce S.K. (1993). Cloning of the gene encoding peptide-binding protein 74 shows that it is a new member of the heat shock protein 70 family. Mol. Cell. Biol..

[4] Webster T.J., Naylor D.J., Hartman D.J., Hoj P.B., Hoogenraad N.J. (1994). cDNA cloning and efficient mitochondrial import of pre-mtHSP70 from rat liver. DNA Cell Biol..

[5] Deocaris C.C., Kaul S.C., Wadhwa R. (2006). On the brotherhood of the mitochondrial chaperones mortalin and heat shock protein 60. Cell Stress Chaperones.

[6] Czarnecka A.M., Campanella C., Zummo G., Cappello F. (2006). Mitochondrial chaperones in cancer: From molecular biology to clinical diagnostics. Cancer Biol. Ther..

[7] Dundas S.R., Lawrie L.C., Rooney P.H., Murray G.I. (2005). Mortalin is over-expressed by colorectal adenocarcinomas and correlates with poor survival. J. Pathol..

[8] Ohashi M., Oyanagi M., Hatakeyama K., Inoue M., Kominami R. (1995). The gene encoding PBP74/CSA/motalin-1, a novel mouse hsp70, maps to mouse chromosome 18. Genomics.

[9] Wadhwa R., Takano S., Kaur K., Aida S., Yaguchi T., Kaul Z., Hirano T., Taira K., Kaul S.C. (2005). Identification and characterization of molecular interactions between mortalin/mthsp70 and hsp60. Biochem. J..

[10] Xie H., Hu Z., Chyna B., Horrigan S.K., Westbrook C.A. (2000). Human mortalin (HSPA9): A candidate for the myeloid leukemia tumor suppressor gene on 5q31. Leukemia.

[11] Deocaris C.C., Kaul S.C., Wadhwa R. (2009). The versatile stress protein mortalin as a chaperone therapeutic agent. Protein Pept. Lett..

[12] Wadhwa R., Taira K., Kaul S.C. (2002). An Hsp70 family chaperone, mortalin/mthsp70/PBP74/Grp75: What, when, and where?. Cell Stress Chaperones.

[13] Daugaard M., Rohde M., Jaattela M. (2007). The heat shock protein 70 family: Highly homologous proteins with overlapping and distinct functions. FEBS Lett..

[14] Iosefson O., Sharon S., Goloubinoff P., Azem A. (2012). Reactivation of protein aggregates by mortalin and Tid1-the human mitochondrial Hsp70 chaperone system. Cell Stress Chaperones.

[15] Yang H., Zhou X., Liu X., Yang L., Chen Q., Zhao D., Zuo J., Liu W. (2011). Mitochondrial dysfunction induced by knockdown of mortalin is rescued by Parkin. Biochem. Biophys. Res. Commun..

[16] Yokoyama K., Fukumoto K., Murakami T., Harada S., Hosono R., Wadhwa R., Mitsui Y., Ohkuma S. (2002). Extended longevity of Caenorhabditis elegans by knocking in extra copies of hsp70F, a homolog of mot-2 (mortalin)/mthsp70/Grp75. FEBS Lett..

[17] Ran Q., Wadhwa R., Kawai R., Kaul S.C., Sifers R.N., Bick R.J., Smith J.R., Pereira-Smith O.M. (2000). Extramitochondrial localization of mortalin/mthsp70/PBP74/GRP75. Biochem. Biophys. Res. Commun..

[18] Wadhwa R., Ando H., Kawasaki H., Taira K., Kaul S.C. (2003). Targeting mortalin using conventional and RNA-helicase-coupled hammerhead ribozymes. EMBO Rep..

[19] Deocaris C.C., Kaul S.C., Wadhwa R. (2008). From proliferative to neurological role of an hsp70 stress chaperone, mortalin. Biogerontology.

[20] Osorio C., Sullivan P.M., He D.N., Mace B.E., Ervin J.F., Strittmatter W.J., Alzate O. (2007). Mortalin is regulated by APOE in hippocampus of AD patients and by human APOE in TR mice. Neurobiol. Aging.

[21] DeKroon R.M., Osorio C., Robinette J.B., Mocanu M., Winnik W.M., Alzate O. (2011). Simultaneous detection of changes in protein expression and oxidative modification as a function of age and APOE genotype. J. Proteome Res..

[22] The PyMOL Molecular Graphics System, Version 1.2r3pre, Schrödinger, LLC. http://www.pymol.org/.

[23] Iosefson O., Azem A. (2010). Reconstitution of the mitochondrial Hsp70 (mortalin)-p53 interaction using purified proteins—Identification of additional interacting regions. FEBS Lett..

[24] Liu Y., Liu W., Song X.D., Zuo J. (2005). Effect of GRP75/mthsp70/PBP74/mortalin overexpression on intracellular ATP level, mitochondrial membrane potential and ROS accumulation following glucose deprivation in PC12 cells. Mol. Cell. Biochem..

[25] Merrick B.A., Walker V.R., He C., Patterson R.M., Selkirk J.K. (1997). Induction of novel Grp75 isoforms by 2-deoxyglucose in human and murine fibroblasts. Cancer Lett..

[26] Resendez E., Attenello J.W., Grafsky A., Chang C.S., Lee A.S. (1985). Calcium ionophore A23187 induces expression of glucose-regulated genes and their heterologous fusion genes. Mol. Cell. Biol..

[27] Craig E.E., Chesley A., Hood D.A. (1998). Thyroid hormone modifies mitochondrial phenotype by increasing protein import without altering degradation. Am. J. Physiol..

[28] Sadekova S., Lehnert S., Chow T.Y. (1997). Induction of PBP74/mortalin/Grp75, a member of the hsp70 family, by low doses of ionizing radiation: A possible role in induced radioresistance. Int. J. Radiat. Biol..

[29] Kaul S.C., Reddel R.R., Sugihara T., Mitsui Y., Wadhwa R. (2000). Inactivation of p53 and life span extension of human diploid fibroblasts by mot-2. FEBS Lett..

[30] Qu M., Zhou Z., Xu S., Chen C., Yu Z., Wang D. (2011). Mortalin overexpression attenuates beta-amyloid-induced neurotoxicity in SH-SY5Y cells. Brain Res..

[31] Xu L., Voloboueva L.A., Ouyang Y., Emery J.F., Giffard R.G. (2009). Overexpression of mitochondrial Hsp70/Hsp75 in rat brain protects mitochondria, reduces oxidative stress, and protects from focal ischemia. J. Cereb. Blood Flow Metab..

[32] Taurin S., Seyrantepe V., Orlov S.N., Tremblay T.L., Thibault P., Bennett M.R., Hamet P., Pshezhetsky A.V. (2002). Proteome analysis and functional expression identify mortalin as an antiapoptotic gene induced by elevation of [Na+]i/[K+]i ratio in cultured vascular smooth muscle cells. Circ. Res..

[33] Kaul S.C., Yaguchi T., Taira K., Reddel R.R., Wadhwa R. (2003). Overexpressed mortalin (mot-2)/mthsp70/GRP75 and hTERT cooperate to extend the *in vitro* lifespan of human fibroblasts. Exp. Cell Res..

[34] Kaul S.C., Deocaris C.C., Wadhwa R. (2007). Three faces of mortalin: A housekeeper, guardian and killer. Exp. Gerontol..

[35] Mizukoshi E., Suzuki M., Loupatov A., Uruno T., Hayashi H., Misono T., Kaul S.C., Wadhwa R., Imamura T. (1999). Fibroblast growth factor-1 interacts with the glucose-regulated protein GRP75/mortalin. Biochem. J..

[36] Sacht G., Brigelius-Flohe R., Kiess M., Sztajer H., Flohe L. (1999). ATP-sensitive association of mortalin with the IL-1 receptor type I. Biofactors.

[37] Pilzer D., Fishelson Z. (2005). Mortalin/GRP75 promotes release of membrane vesicles from immune attacked cells and protection from complement-mediated lysis. Int. Immunol..

[38] Carette J., Lehnert S., Chow T.Y. (2002). Implication of PBP74/mortalin/GRP75 in the radio-adaptive response. Int. J. Radiat. Biol..

[39] Ibi T., Sahashi K., Ling J., Marui K., Mitsuma T. (1996). Immunostaining of mitochondrial heat shock proteins (mtHSPs) in skeletal muscle fibers of mitochondrial cytopathy. Rinsho Shinkeigaku.

[40] Rivolta M.N., Holley M.C. (2002). Asymmetric segregation of mitochondria and mortalin correlates with the multi-lineage potential of inner ear sensory cell progenitors *in vitro*. Brain Res. Dev. Brain Res..

[41] Kaul S.C., Taira K., Pereira-Smith O.M., Wadhwa R. (2002). Mortalin: Present and prospective. Exp. Gerontol..

[42] Wadhwa R., Yaguchi T., Hasan M.K., Mitsui Y., Reddel R.R., Kaul S.C. (2002). Hsp70 family member, mot-2/mthsp70/GRP75, binds to the cytoplasmic sequestration domain of the p53 protein. Exp. Cell Res..

[43] Kogel D., Schomburg R., Copanaki E., Prehn J.H. (2005). Regulation of gene expression by the amyloid precursor protein: Inhibition of the JNK/c-Jun pathway. Cell Death Differ..

[44] Feng Y., Ariza M.E., Goulet A.C., Shi J., Nelson M.A. (2005). Death-signal-induced relocalization of cyclin-dependent kinase 11 to mitochondria. Biochem. J..

[45] Burbulla L.F., Schelling C., Kato H., Rapaport D., Woitalla D., Schiesling C., Schulte C., Sharma M., Illig T., Bauer P. (2010). Dissecting the role of the mitochondrial chaperone mortalin in Parkinson’s disease: Functional impact of disease-related variants on mitochondrial homeostasis. Hum. Mol. Genet..

[46] Canet-Aviles R.M., Wilson M.A., Miller D.W., Ahmad R., McLendon C., Bandyopadhyay S., Baptista M.J., Ringe D., Petsko G.A., Cookson M.R. (2004). The Parkinson’s disease protein DJ-1 is neuroprotective due to cysteine-sulfinic acid-driven mitochondrial localization. Proc. Natl. Acad. Sci. USA.

[47] Jin J., Hulette C., Wang Y., Zhang T., Pan C., Wadhwa R., Zhang J. (2006). Proteomic identification of a stress protein, mortalin/mthsp70/GRP75: Relevance to Parkinson disease. Mol. Cell. Proteomics.

[48] Mizukoshi E., Suzuki M., Misono T., Loupatov A., Munekata E., Kaul S.C., Wadhwa R., Imamura T. (2001). Cell-cycle dependent tyrosine phosphorylation on mortalin regulates its interaction with fibroblast growth factor-1. Biochem. Biophys. Res. Commun..

[49] Takano S., Wadhwa R., Mitsui Y., Kaul S.C. (2001). Identification and characterization of molecular interactions between glucose-regulated proteins (GRPs) mortalin/GRP75/peptide-binding protein 74 (PBP74) and GRP94. Biochem. J..

[50] Kuwabara H., Yoneda M., Hayasaki H., Nakamura T., Mori H. (2006). Glucose regulated proteins 78 and 75 bind to the receptor for hyaluronan mediated motility in interphase microtubules. Biochem. Biophys. Res. Commun..

[51] Wadhwa R., Yaguchi T., Hasan M.K., Taira K., Kaul S.C. (2003). Mortalin-MPD (mevalonate pyrophosphate decarboxylase) interactions and their role in control of cellular proliferation. Biochem. Biophys. Res. Commun..

[52] Wadhwa R., Takano S., Robert M., Yoshida A., Nomura H., Reddel R.R., Mitsui Y., Kaul S.C. (1998). Inactivation of tumor suppressor p53 by mot-2, a hsp70 family member. J. Biol. Chem..

[53] Orsini F., Migliaccio E., Moroni M., Contursi C., Raker V.A., Piccini D., Martin-Padura I., Pelliccia G., Trinei M., Bono M. (2004). The life span determinant p66Shc localizes to mitochondria where it associates with mitochondrial heat shock protein 70 and regulates trans-membrane potential. J. Biol. Chem..

[54] Pellegrini M., Pacini S., Baldari C.T. (2005). p66SHC: The apoptotic side of Shc proteins. Apoptosis.

[55] Grunewald A., Voges L., Rakovic A., Kasten M., Vandebona H., Hemmelmann C., Lohmann K., Orolicki S., Ramirez A., Schapira A.H. (2010). Mutant Parkin impairs mitochondrial function and morphology in human fibroblasts. PLoS One.

[56] Goswami A.V., Chittoor B., D’Silva P. (2010). Understanding the functional interplay between mammalian mitochondrial Hsp70 chaperone machine components. J. Biol. Chem..

[57] Voloboueva L.A., Duan M., Ouyang Y., Emery J.F., Stoy C., Giffard R.G. (2008). Overexpression of mitochondrial Hsp70/Hsp75 protects astrocytes against ischemic injury *in vitro*. J. Cereb. Blood Flow Metab..

[58] Schwarzer C., Barnikol-Watanabe S., Thinnes F.P., Hilschmann N. (2002). Voltage-dependent anion-selective channel (VDAC) interacts with the dynein light chain Tctex1 and the heat-shock protein PBP74. Int. J. Biochem. Cell Biol..

[59] Qu M., Zhou Z., Chen C., Li M., Pei L., Yang J., Wang Y., Li L., Liu C., Zhang G. (2012). Inhibition of mitochondrial permeability transition pore opening is involved in the protective effects of mortalin overexpression against beta-amyloid-induced apoptosis in SH-SY5Y cells. Neurosci. Res..

[60] Ornatsky O.I., Connor M.K., Hood D.A. (1995). Expression of stress proteins and mitochondrial chaperonins in chronically stimulated skeletal muscle. Biochem. J..

[61] Takahashi M., Chesley A., Freyssenet D., Hood D.A. (1998). Contractile activity-induced adaptations in the mitochondrial protein import system. Am. J. Physiol..

[62] Brunner M., Schneider H.C., Lill R., Neupert W. (1995). Dissection of protein translocation across the mitochondrial outer and inner membranes. Cold Spring Harb. Symp. Quant. Biol..

[63] Schneider H.C., Berthold J., Bauer M.F., Dietmeier K., Guiard B., Brunner M., Neupert W. (1994). Mitochondrial Hsp70/MIM44 complex facilitates protein import. Nature.

[64] Voos W. (2009). Mitochondrial protein homeostasis: The cooperative roles of chaperones and proteases. Res. Microbiol..

[65] Voos W., Martin H., Krimmer T., Pfanner N. (1999). Mechanisms of protein translocation into mitochondria. Biochim. Biophys. Acta.

[66] Harner M., Neupert W., Deponte M. (2011). Lateral release of proteins from the TOM complex into the outer membrane of mitochondria. EMBO J..

[67] Voos W., Rottgers K. (2002). Molecular chaperones as essential mediators of mitochondrial biogenesis. Biochim. Biophys. Acta.

[68] Lim J.H., Martin F., Guiard B., Pfanner N., Voos W. (2001). The mitochondrial Hsp70-dependent import system actively unfolds preproteins and shortens the lag phase of translocation. EMBO J..

[69] Marom M., Azem A., Mokranjac D. (2011). Understanding the molecular mechanism of protein translocation across the mitochondrial inner membrane: Still a long way to go. Biochim. Biophys. Acta.

[70] Neupert W., Brunner M. (2002). The protein import motor of mitochondria. Nat. Rev. Mol. Cell. Biol..

[71] Scherer P.E., Manning-Krieg U.C., Jeno P., Schatz G., Horst M. (1992). Identification of a 45-kDa protein at the protein import site of the yeast mitochondrial inner membrane. Proc. Natl. Acad. Sci. USA.

[72] D’Silva P., Liu Q., Walter W., Craig E.A. (2004). Regulated interactions of mtHsp70 with Tim44 at the translocon in the mitochondrial inner membrane. Nat. Struct. Mol. Biol..

[73] Elsner S., Simian D., Iosefson O., Marom M., Azem A. (2009). The mitochondrial protein translocation motor: Structural conservation between the human and yeast Tim14/Pam18-Tim16/Pam16 co-chaperones. Int. J. Mol. Sci..

[74] Mokranjac D., Sichting M., Neupert W., Hell K. (2003). Tim14, a novel key component of the import motor of the TIM23 protein translocase of mitochondria. EMBO J..

[75] Sastry P.S., Rao K.S. (2000). Apoptosis and the nervous system. J. Neurochem..

[76] Reed T.T., Sultana R., Butterfield D.A., Alzate O. (2010). Redox Proteomics of Oxidatively Modified Brain Proteins in Mild Cognitive Impairmen. Neuroproteomics.

[77] Kimura K., Tanaka N., Nakamura N., Takano S., Ohkuma S. (2007). Knockdown of mitochondrial heat shock protein 70 promotes progeria-like phenotypes in caenorhabditis elegans. J. Biol. Chem..

[78] Chipuk J.E., Green D.R. (2008). How do BCL-2 proteins induce mitochondrial outer membrane permeabilization?. Trends Cell Biol..

[79] Danial N.N., Korsmeyer S.J. (2004). Cell death: Critical control points. Cell.

[80] Strasser A., Cory S., Adams J.M. (2011). Deciphering the rules of programmed cell death to improve therapy of cancer and other diseases. EMBO J..

[81] Deshmukh M., Johnson E.M. (2000). Staurosporine-induced neuronal death: Multiple mechanisms and methodological implications. Cell Death Differ..

[82] Fuchs Y., Steller H. (2011). Programmed cell death in animal development and disease. Cell.

[83] Woo M., Hakem R., Mak T.W. (2000). Executionary pathway for apoptosis: Lessons from mutant mice. Cell Res..

[84] Bratton S.B., Salvesen G.S. (2010). Regulation of the Apaf-1-caspase-9 apoptosome. J. Cell Sci..

[85] Chandra J., Samali A., Orrenius S. (2000). Triggering and modulation of apoptosis by oxidative stress. Free Radic. Biol. Med..

[86] Llambi F., Green D.R. (2011). Apoptosis and oncogenesis: Give and take in the BCL-2 family. Curr. Opin. Genet. Dev..

[87] Llambi F., Moldoveanu T., Tait S.W., Bouchier-Hayes L., Temirov J., McCormick L.L., Dillon C.P., Green D.R. (2011). A unified model of mammalian BCL-2 protein family interactions at the mitochondria. Mol. Cell.

[88] Riedl S.J., Li W., Chao Y., Schwarzenbacher R., Shi Y. (2005). Structure of the apoptotic protease-activating factor 1 bound to ADP. Nature.

[89] Zou H., Henzel W.J., Liu X., Lutschg A., Wang X. (1997). Apaf-1, a human protein homologous to C. elegans CED-4, participates in cytochrome c-dependent activation of caspase-3. Cell.

[90] Beere H.M., Wolf B.B., Cain K., Mosser D.D., Mahboubi A., Kuwana T., Tailor P., Morimoto R.I., Cohen G.M., Green D.R. (2000). Heat-shock protein 70 inhibits apoptosis by preventing recruitment of procaspase-9 to the Apaf-1 apoptosome. Nat. Cell Biol..

[91] Ott M., Robertson J.D., Gogvadze V., Zhivotovsky B., Orrenius S. (2002). Cytochrome c release from mitochondria proceeds by a two-step process. Proc. Natl. Acad. Sci. USA.

[92] Lu W.J., Lee N.P., Kaul S.C., Lan F., Poon R.T., Wadhwa R., Luk J.M. (2011). Mortalin-p53 interaction in cancer cells is stress dependent and constitutes a selective target for cancer therapy. Cell Death Differ..

[93] Zhang H., Reed J.C. (2001). Studies of apoptosis proteins in yeast. Methods Cell Biol..

[94] Liu S., Li J., Tao Y., Xiao X. (2007). Small heat shock protein alphaB-crystallin binds to p53 to sequester its translocation to mitochondria during hydrogen peroxide-induced apoptosis. Biochem. Biophys. Res. Commun..

[95] Nikolaev A.Y., Li M., Puskas N., Qin J., Gu W. (2003). Parc: A cytoplasmic anchor for p53. Cell.

[96] Zhao L.Y., Liao D. (2003). Sequestration of p53 in the cytoplasm by adenovirus type 12 E1B 55-kilodalton oncoprotein is required for inhibition of p53-mediated apoptosis. J. Virol..

[97] Zilfou J.T., Lowe S.W. (2009). Tumor suppressive functions of p53. Cold Spring Harb. Perspect. Biol..

[98] Deocaris C.C., Widodo N., Ishii T., Kaul S.C., Wadhwa R. (2007). Functional significance of minor structural and expression changes in stress chaperone mortalin. Ann. N. Y. Acad. Sci..

[99] Kaul S.C., Aida S., Yaguchi T., Kaur K., Wadhwa R. (2005). Activation of wild type p53 function by its mortalin-binding, cytoplasmically localizing carboxyl terminus peptides. J. Biol. Chem..

[100] Liu B., Chen Y., St Clair D.K. (2008). ROS and p53: A versatile partnership. Free Radic. Biol. Med..

[101] Soti C., Csermely P. (2002). Chaperones and aging: Role in neurodegeneration and in other civilizational diseases. Neurochem. Int..

[102] Berlett B.S., Stadtman E.R. (1997). Protein oxidation in aging, disease, and oxidative stres. J. Biol. Chem..

[103] Choi J., Forster M.J., McDonald S.R., Weintraub S.T., Carroll C.A., Gracy R.W. (2004). Proteomic identification of specific oxidized proteins in ApoE-knockout mice: Relevance to Alzheimer’s disease. Free Radic. Biol. Med..

[104] Yaguchi T., Aida S., Kaul S.C., Wadhwa R. (2007). Involvement of mortalin in cellular senescence from the perspective of its mitochondrial import, chaperone, and oxidative stress management function. Ann. N. Y. Acad. Sci..

[105] Mahley R.W., Weisgraber K.H., Huang Y. (2006). Apolipoprotein E4: A causative factor and therapeutic target in neuropathology, including Alzheimer’s disease. Proc. Natl. Acad. Sci. USA.

[106] Saunders A.M., Strittmatter W.J., Schmechel D., George-Hyslop P.H., Pericak-Vance M.A., Joo S.H., Rosi B.L., Gusella J.F., Crapper-MacLachlan D.R., Alberts M.J. (1993). Association of apolipoprotein E allele epsilon 4 with late-onset familial and sporadic Alzheimer’s disease. Neurology.

[107] Strittmatter W.J., Saunders A.M., Schmechel D., Pericak-Vance M., Enghild J., Salvesen G.S., Roses A.D. (1993). Apolipoprotein E: High-avidity binding to beta-amyloid and increased frequency of type 4 allele in late-onset familial Alzheimer disease. Proc. Natl. Acad. Sci. USA.

[108] Strittmatter W.J., Weisgraber K.H., Huang D.Y., Dong L.M., Salvesen G.S., Pericak-Vance M., Schmechel D., Saunders A.M., Goldgaber D., Roses A.D. (1993). Binding of human apolipoprotein E to synthetic amyloid beta peptide: Isoform-specific effects and implications for late-onset Alzheimer disease. Proc. Natl. Acad. Sci. USA.

[109] Beal M.F. (1995). Aging, energy, and oxidative stress in neurodegenerative disease. Ann. Neurol..

[110] McNaught K.S., Olanow C.W., Halliwell B., Isacson O., Jenner P. (2001). Failure of the ubiquitin-proteasome system in Parkinson’s disease. Nat. Rev. Neurosci..

[111] Youle R.J., Narendra D.P. (2011). Mechanisms of mitophagy. Nat. Rev. Mol. Cell. Biol..

[112] Samii A., Nutt J.G., Ransom B.R. (2004). Parkinson’s disease. Lancet.

[113] Van Laar V.S., Dukes A.A., Cascio M., Hastings T.G. (2008). Proteomic analysis of rat brain mitochondria following exposure to dopamine quinone: Implications for Parkinson disease. Neurobiol. Dis..

[114] Tieu K., Ischiropoulos H., Przedborski S. (2003). Nitric oxide and reactive oxygen species in Parkinson’s disease. IUBMB Life.

[115] Geissler A., Rassow J., Pfanner N., Voos W. (2001). Mitochondrial import driving forces: Enhanced trapping by matrix Hsp70 stimulates translocation and reduces the membrane potential dependence of loosely folded preproteins. Mol. Cell. Biol..

[116] Narendra D., Kane L.A., Hauser D.N., Fearnley I.M., Youle R.J. (2010). p62/SQSTM1 is required for Parkin-induced mitochondrial clustering but not mitophagy; VDAC1 is dispensable for both. Autophagy.

[117] Malgieri G., Eliezer D. (2008). Structural effects of Parkinson’s disease linked DJ-1 mutations. Protein Sci..

[118] Li H.M., Niki T., Taira T., Iguchi-Ariga S.M., Ariga H. (2005). Association of DJ-1 with chaperones and enhanced association and colocalization with mitochondrial Hsp70 by oxidative stress. Free Radic. Res..

[119] Mattson M.P. (2000). Apoptosis in neurodegenerative disorders. Nat. Rev. Mol. Cell. Biol..

[120] Oppenheim R.W. (1991). Cell death during development of the nervous system. Annu. Rev. Neurosci..

[121] Mattson M.P. (2004). Metal-catalyzed disruption of membrane protein and lipid signaling in the pathogenesis of neurodegenerative disorders. Ann. N. Y. Acad. Sci..

